# Laboratory Grown Biofilms of Bacteria Associated with Human Atherosclerotic Carotid Arteries Release Collagenases and Gelatinases during Iron-Induced Dispersion

**DOI:** 10.1128/spectrum.01001-21

**Published:** 2022-05-11

**Authors:** Amanda M. Zdimal, David G. Davies

**Affiliations:** a Department of Biological Sciences and Binghamton Biofilm Research Center, Binghamton University, Binghamton, New York, USA; University of Guelph

**Keywords:** biofilm, dispersion, atherosclerosis

## Abstract

The association of bacteria with arterial plaque lesions in patients with atherosclerosis has been widely reported. However, the role these bacteria play in the progression of atherosclerosis is still unclear. Previous work in our lab has demonstrated that bacteria exist in carotid artery plaques as biofilm deposits. Biofilms are communities of microorganisms enmeshed within a protective, self-produced extracellular matrix and have been shown to contribute to chronic infections in humans. Biofilm communities have the potential to impact surrounding tissues in an infection if they undergo a dispersion response, releasing bacteria into the surrounding environment by enzymatic degradation of the extracellular matrix. One concern relating to these enzymes is that they could cause collateral damage to host tissues. In this study, we present an *in vitro* multispecies biofilm culturing model used to investigate the potential role of bacterial biofilm dispersion in the progression of atherosclerosis. This work has demonstrated an increase in cell release from mixed-species biofilms formed by bacteria associated with human carotid arterial plaque deposits following treatment with iron or a combination of norepinephrine and transferrin. Greater extracellular lipase, protease, and collagenase/gelatinase activity was also associated with iron-treated biofilms. The results of this work suggest that bacteria in this model undergo iron-induced biofilm dispersion, as evidenced by the increased cell release and higher enzyme activity following treatment. This work demonstrates the potential for multispecies biofilm dispersion to contribute to arterial tissue degradation by bacteria and suggests that in atherosclerotic infections, biofilm dispersion may contribute to thrombogenesis, which can lead to heart attack or stroke.

**IMPORTANCE** Atherosclerosis, or hardening of the arteries, is a leading cause of congestive heart failure, heart attack, and stroke in humans. Mounting evidence, in the literature and from our lab, points to the regular involvement of bacteria within arterial plaque deposits in patients with advanced atherosclerosis. Very little is known about the behavior of these bacteria and whether they may contribute to tissue damage in infected arteries. Tissue damage within the arterial plaque lesion can lead to rupture of the plaque contents into the bloodstream, where a clot may form, resulting in a potential heart attack or stroke. This study shows that plaque-associated bacteria, when cultured as mixed-species biofilms in the laboratory, can release degradative enzymes into their environment as the result of a dispersion response triggered by iron. These degradative enzymes can digest proteins and lipids which are associated with the tissues that separate the plaque lesion from the arterial lumen. Thus, this study demonstrates that if mixed species biofilms are induced to undergo dispersion in an infected atherosclerotic lesion when exposed to an elevated concentration of free iron, they have the potential to contribute to the weakening of arterial tissues, which may contribute to atherosclerotic plaque destabilization.

## INTRODUCTION

Atherosclerosis is a chronic, progressive, inflammatory disease characterized by hardening of the arterial walls. This disease is a principal underlying cause of heart attack and stroke, which have remained the leading causes of death in humans worldwide for over 20 years (1). Atherosclerosis has been traditionally understood to be driven by an elevated level of low-density lipoprotein (LDL) cholesterol, which is deposited within arterial walls where it may activate several pathways that promote atherogenesis (2). Over time, the biology of the plaque changes and, in many patients, it transitions to an unstable state characterized by a large necrotic lipid core infiltrated by macrophages and neutrophils, reduction of collagen and smooth muscle cells, and the development of a thin fibrous cap (3, 4). The fibrous cap is rich in collagen and functions as an endothelial boundary separating the plaque deposit from the arterial lumen. The thinning of this structure contributes to the instability of the plaque deposit and can lead to rupture, resulting in thrombogenesis and risk of heart attack or stroke (5, 6).

Bacterial 16S rRNA genes have been identified in atherosclerotic plaque deposits, suggesting the presence of infecting bacteria which may have the potential to influence plaque stability and contribute to the deterioration of the fibrous cap (7–13). Bacterial infections have been associated with a number of cardiovascular diseases, including but not limited to endocarditis (14), cardiomyopathy (15), rheumatic heart disease (16), and pericarditis (17). These associations indicate that bacterial infections may play an influential role in determining cardiovascular health. The role of bacteria in atherosclerosis is not well understood, but several studies have identified diverse bacterial 16S rRNA genes in the aorta as well as in carotid, coronary, femoral, iliac, internal mammary, and pulmonary human arteries (7–13, 18, 19). Prior work in our lab, examining 16S rRNA gene sequences in carotid artery explants from 5 patients with advanced atherosclerosis, has detected between 11 and 19 distinct bacterial taxonomic groups per artery (12). Further study in our lab has shown specific 16S rRNA gene sequences in carotid explants for Cutibacterium acnes (formerly Propionibacterium acnes) in 9 out of 20 patients (13) and Pseudomonas aeruginosa in 6 out of 15 patients (12), with both organisms recoverable in culture from at least one patient sample. We have also recovered two distinct strains of Staphylococcus epidermidis in culture from carotid artery explants from two patients (13). Additionally, our lab was the first to demonstrate that bacteria associated with atheromatous deposits in human carotid arteries exist as biofilm deposits (12).

A biofilm is a community of microorganisms encased in a self-produced extracellular matrix (20). Many pathogens can form biofilms and they are associated with chronic infections in humans. Research has demonstrated that biofilms composed of P. aeruginosa release virulence factors, such as toxins (21) and proteases (22), that damage host tissues and impair immune defenses. Biofilm infections pose additional risks to the host due to their reduced susceptibility to antimicrobials (23) and ability to compete with the host for oxygen and nutrients. One aspect of biofilm behavior that may contribute to pathogenesis is the dispersion response, which results in the active release of bacteria from within a biofilm. Biofilm dispersion is facilitated by the activity of extracellular enzymes which degrade the biofilm matrix (24–28). Little is known about biofilm dispersion, and most studies of this important behavioral aspect of biofilms have been conducted by experimentation using single-species biofilms. Biofilms in nature, however, are typically polymicrobial (29), and while this is likely also true of biofilms in human infections, our understanding of the behavior of mixed species biofilms is very limited.

Stress has been historically correlated with heart attack or stroke, yet the underlying mechanism behind this relationship is not known. It has been demonstrated that once the fight-or-flight hormone, norepinephrine (NE), is released into the bloodstream, it interacts with transferrin (Tf), an iron chelating protein (30), resulting in the release of free iron from Tf (31). Iron is an essential nutrient for bacteria, and the action of iron chelators renders the blood relatively void of free iron (32). It has been shown that a sudden increase in iron availability in an iron-limiting system induces biofilm dispersion in Escherichia coli biofilms (33). Our lab has also shown biofilm dispersion in single-species P. aeruginosa and C. acnes biofilms when they are challenged with physiologic concentrations of iron or NE and Tf (12, 13). We have hypothesized that a sudden increase in free iron may cause biofilm bacteria in arterial plaques to initiate a dispersion response, resulting in the release of enzymes to degrade the biofilm matrix. This is relevant because these enzymes could have the potential to cause collateral damage to the fibrous cap and contribute to plaque rupture and thrombogenesis in patients with advanced atherosclerosis. To address this concern, we developed a 24-well plate *in vitro* biofilm model using *C. acnes*, S. epidermidis, and P. aeruginosa, grew the biofilms to maturity, and challenged them with either iron or NE plus Tf. We assessed cell release using viable plate counts of bacteria present in the liquid overlaying the biofilm, and cells that remained in the biofilm following treatment. The cell-free spent media of the iron- and medium-treated control biofilms were applied to tributyrin and casein substrates to investigate lipase and protease activity. The presence of collagenases and gelatinases could indicate a potential mechanism for bacteria-driven weakening of the fibrous cap. Collagenase and gelatinase activity in the cell-free spent medium following iron-induced dispersion were quantified using DQ Gelatin, and cell-free medium-only treated biofilm supernatant liquids were used as a control. The findings of this study showed significant biofilm cell release upon the addition of iron, accompanied by the release of lipases and proteases, including collagenases and gelatinases. These results demonstrated that multispecies biofilm dispersion was inducible when bacteria experienced a sudden increase in free iron in their environment.

## RESULTS

### Biofilm growth and development.

We have demonstrated the ability to grow three organisms, *C. acnes*, S. epidermidis, and P. aeruginosa, commonly detected by our lab in carotid atherosclerotic plaque deposits together in a biofilm (referred to as three-species biofilm). Due to high levels of competition between organisms in three-species biofilms, we excluded any biofilm dispersion replicates when the relative abundance of P. aeruginosa in the biofilm was less than 10^5^ cells. This threshold level was chosen because P. aeruginosa was inoculated with approximately 5 × 10^5^ cells, therefore, if it did not maintain that level of survivability, a stable consortium of all three species had not been reached within the biofilm. Both *C. acnes* and S. epidermidis reached consistently high concentrations (between 10^7^ and 10^8^ cells) in all replicates. Biofilm development was achieved with similar consistency across technical and biological replicates (Fig. S1 in the supplemental material).

### Multispecies dispersion.

Most studies on biofilm dispersion have been performed in single-species biofilms, and little is known about multispecies biofilms. Bacterial biofilms present in carotid arterial plaque deposits have been shown to contain multiple species (13). Therefore, we developed a model using three organisms commonly detected by our lab in carotid arterial plaque deposits to provide a more accurate representation of the microbial community structure associated with atherosclerosis. This model was used to investigate whether cell release from the biofilm occurs following an increase in iron availability, both within the overall community and on a species-specific level. We chose to use iron as a dispersion inducer due to its relevance in the physiological stress response and the fact that it is an essential nutrient for bacteria. An increase in a limiting nutrient has been demonstrated to induce biofilm dispersion in several organisms (34, 35).

**FIG 1:**
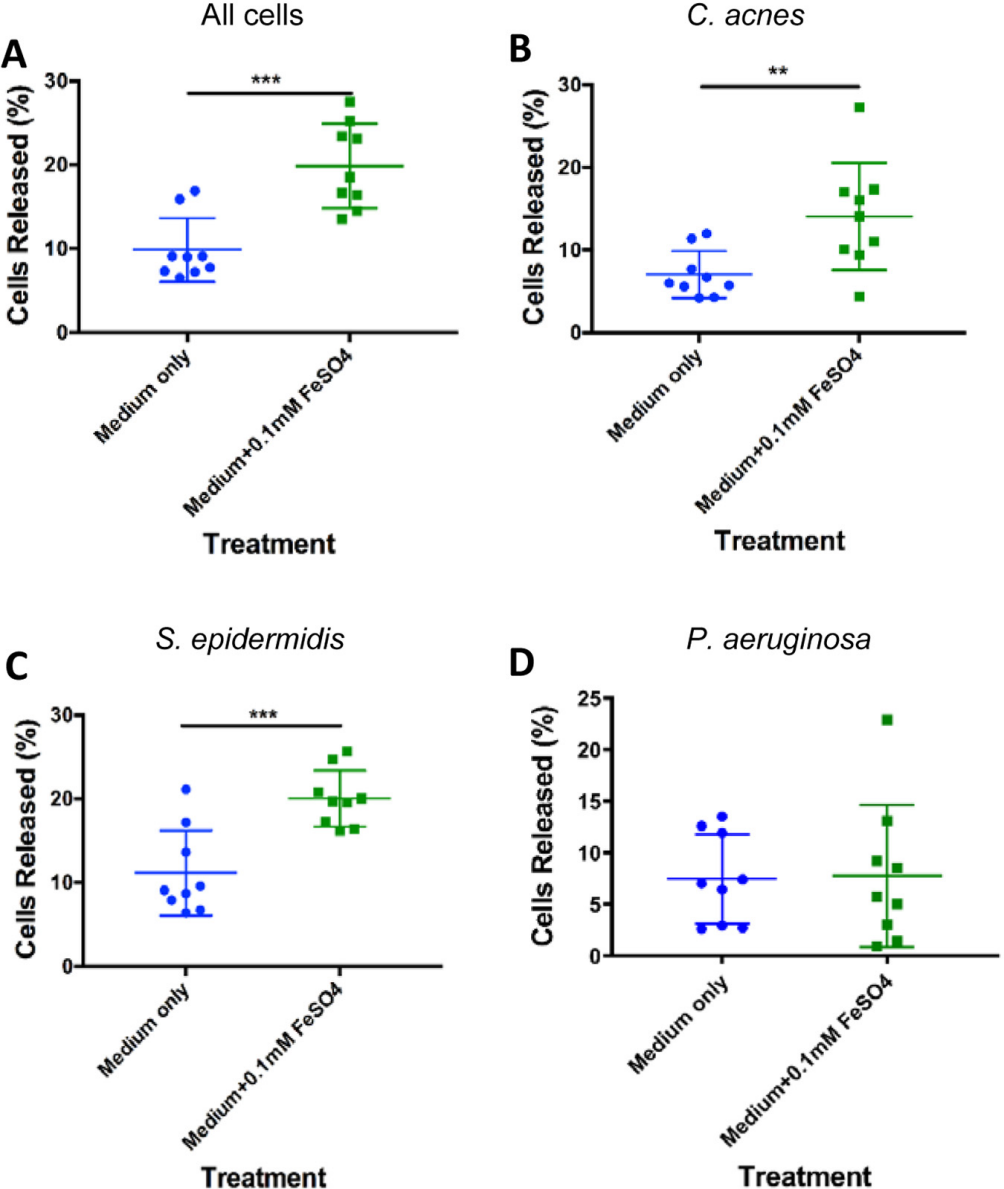
Biofilm dispersion following FeSO4 treatment. Biofilms composed of *C. acnes*, S. epidermidis, and P. aeruginosa (referred to as tri-species biofilms) were grown for a period of 5 days and treated with either 0.1 mM FeSO4 dissolved in 1/5 reinforced clostridial medium (RCM) + 0.1 mM FeSO4, or RCM medium alone as a control. Data represent the percentage of cells present in the supernatant liquid compared to the total number of cells in the supernatant liquid and biofilm. Percent release was calculated for cells from the entire bacterial community as well as for individual populations of *C. acnes*, S. epidermidis, and P. aeruginosa. (A) Percent release of the total number cells in the biofilm community, (B) only *C. acnes* cells, (C) only S. epidermidis cells, and (D) only P. aeruginosa cells. A *t* test was run to determine statistical differences in these data sets (*n* = 3; ***, *P* < 0.001; **, *P* < 0.01; *,*P* < 0.05).

**FIG 2:**
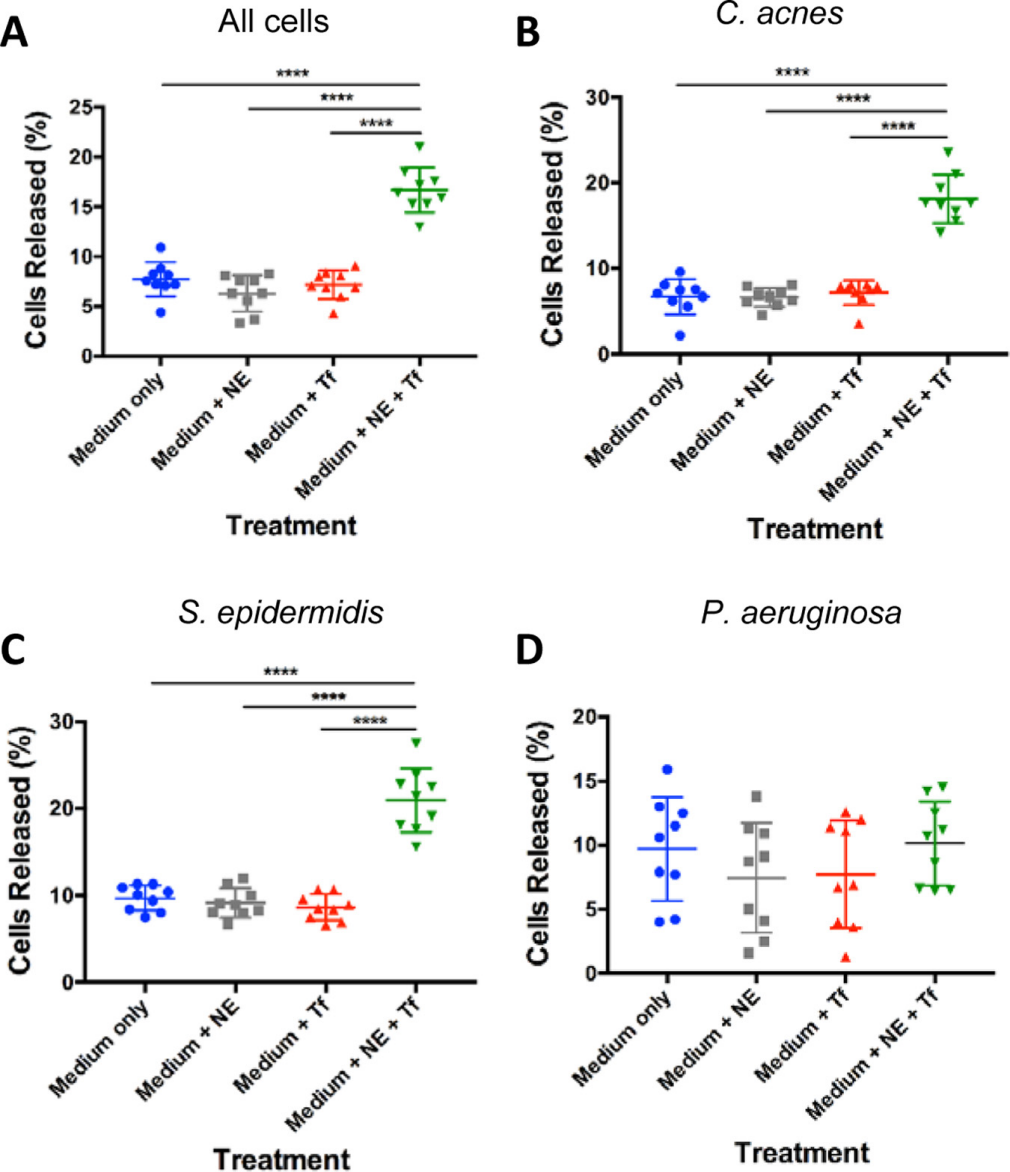
Biofilm dispersion following norepinephrine (NE) + transferrin (Tf) treatment. Five-day biofilms composed of *C. acnes*, S. epidermidis, and P. aeruginosa were treated with 0.5 g/L Tf with 0.4 mM NE in 1/5 RCM (Medium + NE + Tf), 0.5 g/L Tf in 1/5 RCM (Medium + Tf), 0.4 mM NE in 1/5 RCM (Medium + NE) or 1/5 RCM (Medium only). Percent dispersion of cells from (A) all three organisms, (B) only *C. acnes*, only (C) S. epidermidis, and (D) only P. aeruginosa is shown. An analysis of variance was run to assess statistical differences in these data sets (*n* = 3; ***, *P* < 0.001; **, *P* < 0.01; *, *P* < 0.05).

We grew a mixed consortium of the three aforementioned bacterial species together in a biofilm using 24-well plates for a period of 5 days. After this, they were treated with medium supplemented with either 0.1 mM FeSO_4_ (Fig. 1) or 0.4 mM NE with 0.5 g/L Tf (Fig. 2), which are the physiologic concentrations achieved in the bloodstream during stress (30, 36, 37). Treated biofilms were examined for statistically significant cell release following treatment. We used medium only as a control to determine cell release from the sheer force of adding medium to the wells. For biofilms treated with NE + Tf, we used two additional controls. First, we used medium supplemented with NE to confirm that the presence of this hormone alone did not cause an increase in cell release from the biofilm. We also used medium supplemented with Tf, since some bacteria are capable of competing against iron chelators for bound iron; we wanted to ensure that an increase in cell release was a result of the elevated free iron concentration and not the ability of the bacteria to compete for Tf-bound iron. Additionally, we performed growth curves for the individual species with each of the treatments to show that the increase in cells present in the bulk medium was not a result of an increased growth rate of the organisms in the presence of the treatments (Fig. S2).

To our knowledge, this is the first study to demonstrate that dispersion occurs in multispecies biofilms following treatment with iron (Fig. 1 and 2). Biofilms treated with medium only demonstrated an overall 9.9% cell release from the total bacterial community, whereas biofilms treated with 0.1 mM FeSO_4_ showed 19.9% cell release (Fig. 1A). These data demonstrate that the organisms present in the biofilm are more likely to leave the biofilm when there is an increase in iron availability. On a species level, the population of *C. acnes* showed 7.1% cell release in medium-only controls, compared to 14.1% in biofilms treated with iron (Fig. 1B). S. epidermidis showed average cell releases of 11.1% and 20% when treated with medium only and medium supplemented with iron, respectively (Fig. 1C). However, it appears that dispersion may be more prominent in those two populations than in P. aeruginosa under the conditions tested. P. aeruginosa demonstrated no significant difference in cell release when treated with medium only (7.4%) and medium supplemented with 0.1 mM FeSO_4_ (11.1%) (Fig. 1D).

Results were similar in biofilms treated with NE + Tf. Overall, there were 7.7%, 6.3%, and 7.2% cells present in the supernatants of the medium-only, medium + NE, and medium + Tf controls, but 16.7% total cell release in biofilms treated with NE + Tf (Fig. 2A). *C. acnes* demonstrated a significant increase, from 6.7%, 6.6%, and 7.2% release in the controls and 18.1% cell release in NE + Tf-treated biofilms (Fig. 2B). S. epidermidis had 9.7%, 9.2%, and 8.6% cell release in controls and 20.9% release in NE + Tf-treated biofilms (Fig. 2C). P. aeruginosa showed 9.7%, 7.4%, and 7.7% release in the controls, but 10.1% release in NE + Tf-treated biofilms (Fig. 2D). As in the treatment with FeSO_4_, there was no significant additional P. aeruginosa cell release from biofilms treated with medium only, medium + Tf, or medium + NE + Tf.

### Lipase and protease activity of iron-treated biofilms.

The extracellular biofilm matrix imparts several benefits to cells in a biofilm. The exact composition of the extracellular polymeric matrix varies between organisms; however, certain components are almost always present. These include exopolysaccharides, lipids, proteins, and eDNA (38). For biofilm dispersion to occur, bacteria must degrade these components to allow cells to escape and seek out nutrients or colonize a different location. Due to reports of greater enzyme release during biofilm dispersion, we suspected that dispersion would result in increased enzymatic activity compared to that of cells remaining in a sessile state. Lipids and proteins are the main structural components of atheromatous plaque deposits, so we examined whether biofilm dispersion was associated with enhanced lipase and protease activity.

**FIG 3:**
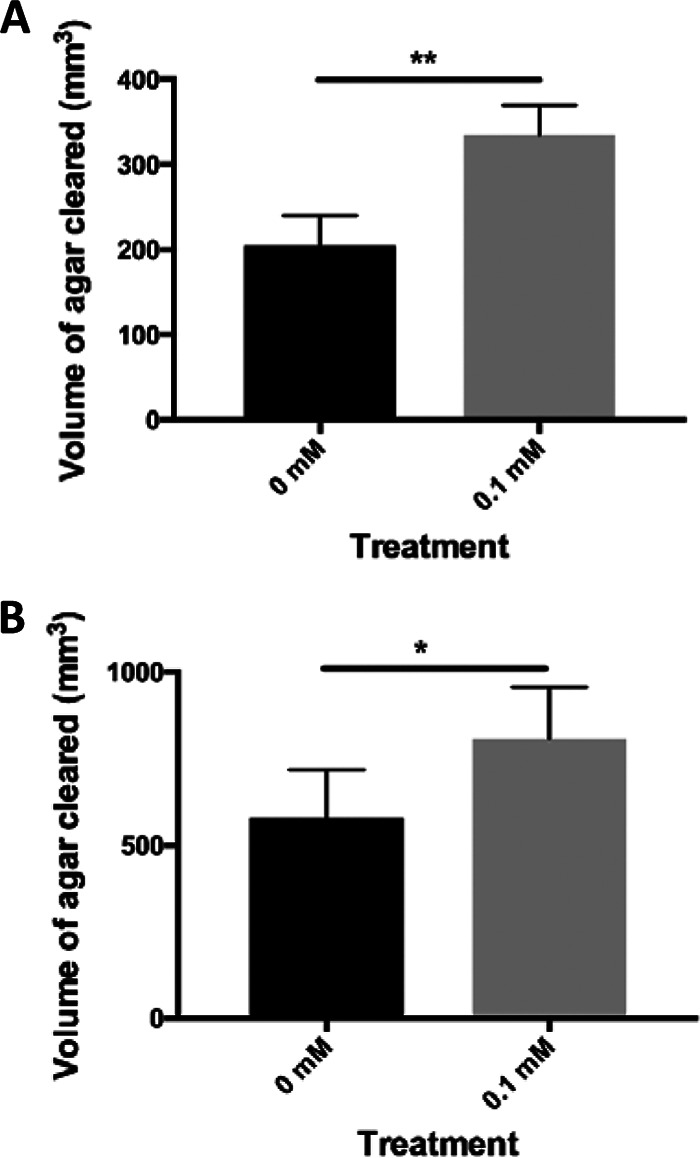
Extracellular lipase and protease activity. (A) Tributyrin agar was used to assess lipase activity of enzymes released by bacterial biofilms treated with iron versus medium only-treated controls. (B) Skim milk agar was used to assess protease activity of enzymes released by iron-dispersed biofilms versus control biofilms. A two-tailed *t* test was used to assess significance between different treatment groups (*n* = 5; *, *P* < 0.05; **, *P* < 0.01). Error bars indicate standard deviations in volume cleared.

The supernatant liquid was collected from FeSO_4_- and medium-treated three-species biofilms, concentrated, and applied to tributyrin agar and skim milk agar. Plates were then incubated, and the diameters of the clearance zones were measured and used along with a measurement of the height of the agar to calculate the volume of agar cleared for each sample. Our data showed greater clearance zones by enzymes present in the supernatant liquids of FeSO_4_-treated biofilms versus medium only-treated biofilms (Fig. 3). These data indicate that greater amounts of active lipases and proteases are released per cell following treatment with iron. Lipase activity in the supernatant liquids of medium-treated control biofilms produced average clearance zones of 579 mm³ in the tributyrin agar and 809 mm³ in dispersed, FeSO_4_-treated biofilms (Fig. 3A). Additionally, protease activity of supernatant liquids demonstrated an average zone of clearing of 204 mm³ in the skim milk agar of medium-treated control biofilms and 334 mm³ in FeSO_4_, dispersed biofilms (Fig. 3B). These data show that all biofilm bacteria release lipases and proteases while in a sessile state, suggesting an ability to alter the integrity of surrounding lipids and proteins when in a biofilm. However, greater amounts of lipases and proteases are released by iron-treated biofilm cells compared to controls, showing that increased cell release is correlated with an increase in lipase and protease activity.

### Gelatinase and collagenase activity of iron-treated biofilms.

We next examined whether particular proteases were released which could damage the fibrous cap. The main structural proteins of the fibrous cap are collagens (39), so we investigated whether greater gelatinase and collagenase activity were detected following treatment with iron and an increase in cell release. Collagenases and gelatinases are released by smooth muscle cells, neutrophils, and macrophages/foam cells in the atheroma, and it is believed that enzymes of human origin are responsible for destabilizing the plaque (40–42). However, the role of bacteria in plaque deposits is not well understood, and we suspect that bacteria may also actively influence the stability of the fibrous cap.

**FIG 4:**
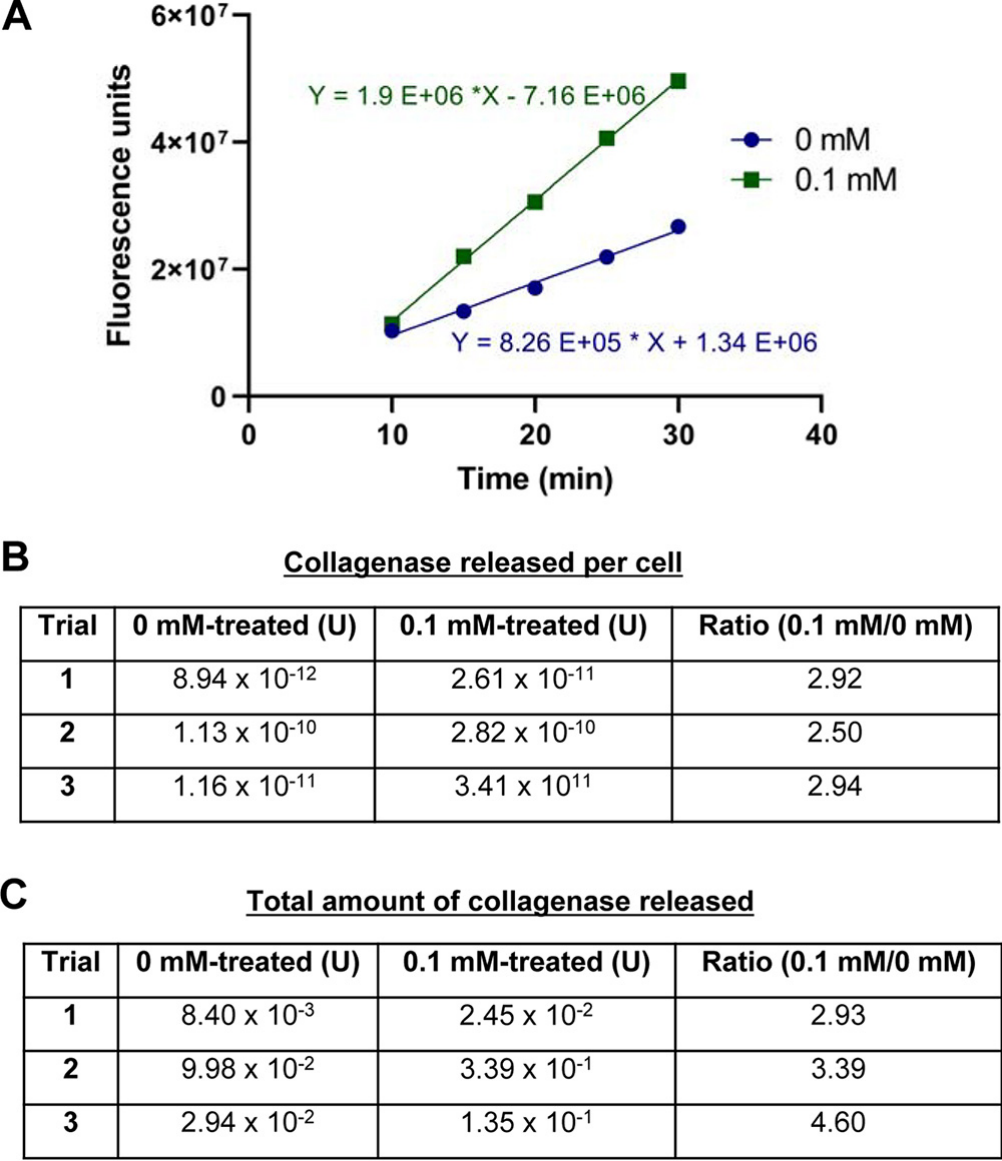
Extracellular collagenase and gelatinase activity. (A) A representative time course from a single experimental trial, demonstrating DQ Gelatin hydrolysis by extracellular enzymes released by biofilm bacteria following treatment with iron. (B) Calculated amounts of enzyme released per cell for each biological replicate. (C) Calculated total amount of collagenase released in each trial (*n* = 3).

DQ Gelatin was used as the substrate for these experiments because it is degraded by essentially all forms of collagenases and gelatinases. The intact substrate is tagged with a fluorophore and quencher in close proximity to one another, and upon proteolytic cleavage the quencher is separated from the fluorophore. Degradation of DQ Gelatin results in fluorescence intensity that is directly proportional to the amount of enzymatic activity acting on the substrate. We combined the DQ Gelatin substrate with the supernatant liquids from the FeSO_4_-treated or medium-treated control biofilms. The data collected from these trials were used to generate enzyme activity curves. The total enzyme concentrations for each replicate were determined, as well as the amount of collagenase released per cell during dispersion or in a biofilm state. We have shown that there is a greater amount of collagenase activity per cell following treatment with iron (Fig. 4). Iron-dispersed cells release between 2.5 and 3 times as much collagenase as their biofilm counterparts (Fig. 4B). The total amount of collagenase present in the dispersed supernatants was approximately 3 to 5 times greater than that of the medium-only controls (Fig. 4C). Our results show that eukaryotic enzymes are potentially not alone in degrading the fibrous cap.

## DISCUSSION

The presence of bacteria in human atheromatous plaques has been reported for over 30 years, yet there is little insight into the physiological role of microorganisms in these locations. Previously, our lab has detected *C. acnes*, S. epidermidis, and P. aeruginosa in human carotid explants. Here, we have successfully developed an *in vitro* three-species biofilm culturing model using *C. acnes* VP1, S. epidermidis ECNU-He1, and P. aeruginosa PA14, which were used as representative community members to model bacteria associated with atherosclerosis. To our knowledge, we are the first to demonstrate iron-induced dispersion in a polymicrobial biofilm, a potential mechanism for cell release and collateral tissue damage within atheromatous lesions. Statistically significant cell release was evident within the overall community (Fig. 1A and Fig. 2A), specifically in the *C. acnes* and S. epidermidis (Fig. 1B and C, Fig. 2B and C) populations. We believe that the increased cell release demonstrated in this study is a result of biofilm dispersion, as it has been shown that a sudden increase in iron availability in an iron-limiting system induces biofilm dispersion (33). Iron in the surrounding environment may act as a nutrient as well as an extracellular signal. In the experiments described here, iron-induced dispersion occurred when the iron level in the medium was suddenly increased. Fig. S2 shows that additional iron did not affect the overall growth rate of the three organisms in the biofilm but did act as a stimulus for biofilm dispersion.

Although there was an observable increase in cell release in P. aeruginosa when it was treated with iron or NE + Tf, these treatments did not show a statistically significant difference from the controls. The lack of increased cell release in response to the addition of iron or NE + Tf could be explained by the fact that P. aeruginosa express siderophores (43–45). The 1/5 reinforced clostridial medium (RCM) used in this study contained low levels of iron, and the action of siderophores may have resulted in P. aeruginosa overcoming iron starvation in this model. *C. acnes* and S. epidermidis are not known to express siderophores, and therefore, these organisms may have experienced iron starvation when grown in the presence of P. aeruginosa. Although P. aeruginosa did not demonstrate evidence of a dispersion response in this model (Fig. 1D and Fig. 2D), a previous study by Lanter et al. (12) demonstrated dispersion in pure-culture biofilms of P. aeruginosa PAO1 in a continuous culture system using minimal medium. The use of different strains and culturing conditions could also account for the differential response following treatment with iron.

Although a sudden increase in free iron has been shown to induce a dispersion response in a single-species biofilm, our findings demonstrated that polymicrobial biofilms also disperse in response to a sudden increase in iron. Biofilms found in nature, including those in atherosclerotic lesions, are polymicrobial, and therefore likely also display this dispersion response. Here, we have shown that two of the three species in the biofilms studied exhibited significant cell release upon the addition of iron. It is possible that not all species or strains respond to iron as a dispersion signal. However, the failure of one organism in a biofilm to disperse did not prevent dispersion by the other organisms in this study. Furthermore, inducing a dispersion response in some but not all bacteria in a polymicrobial biofilm may not result in the release of all cells of the biofilm into the surrounding environment. However, it is possible that enzyme release into the environment during a “failed” dispersion response could still result in damage to surrounding tissues.

Proteins and lipids are essential structural components of connective tissues. The degradation of macromolecules within arterial tissues could have detrimental effects on the stability of atherosclerotic lesions, especially those at risk of rupture. Eukaryotic cells have been shown to release a variety of proteases and lipases which influence the stability of atherosclerotic lesions. Among these are matrix metalloproteases (MMPs), proteases which are responsible for the remodeling of arterial tissues and known to contribute to destabilization of the fibrous cap (40–42). It is not unreasonable to presume that enzymes released by bacteria may further impact the stability of arterial tissues. Our data show enhanced lipase and protease activity by iron-dispersed multispecies biofilm cells compared to that of medium only-treated controls (Fig. 3). It is not clear from this work whether planktonic cells respond to increased iron by releasing degradative enzymes in the manner of biofilm cells. However, the potential for collateral damage due to proteases and lipases would likely be due primarily to biofilm cells as these cells are in close proximity to connective tissues. It is possible that the addition of metal ions, such as iron, may enhance the activity of proteases and lipases. However, we feel this is unlikely since bacterial iron metalloproteases are mainly involved in energy production and not in exoprotease activity (46). Since we demonstrated greater enzyme activity per cell following treatment with iron, these data further support our contention that the cell release observed in the overall bacterial community, and in the *C. acnes* and S. epidermidis populations, is due to biofilm dispersion. Reports have shown that biofilm dispersion is associated with increased enzyme release to break through the biofilm matrix and allow cells to escape (24–28). In the context of atherosclerosis, bacterial protease and lipase activity could pose a significant problem for arterial tissues.

We have also shown three-species biofilms which release gelatinases/collagenases that degrade gelatin, a denatured form of collagen (Fig. 4). Using DQ Gelatin, enzymatic activity from all gelatinases and collagenases produced by the bacteria in this model were detectable. Enzymatic activity was quantified using a standard curve of collagenase type IV, the enzyme that is included in the kit as a positive control. However, it is possible that the bacteria in this model released slightly different concentrations of enzyme than what we reported. This substrate can detect various types of collagenases and gelatinases, which are indistinguishable using this method. It is possible that the enzymes acting against this substrate are gelatinases, since *C. acnes* and P. aeruginosa are gelatinase-positive (47, 48). S. epidermidis is not known to exhibit gelatinase activity, so it is likely that the majority of the collagenase/gelatinase activity observed in this study originated from *C. acnes*, since it showed evidence of a dispersion response whereas P. aeruginosa did not. Nevertheless, we demonstrated 3 to 5 times more gelatinase/collagenase activity in iron-dispersed biofilms compared to that in non-iron-treated biofilms (Fig. 4C). It is important to note that biofilm bacteria can produce gelatinases and collagenases even in a sessile state, suggesting that bacteria present in atherosclerotic lesions may be able to influence the stability of host tissues independent of a dispersion response. However, greater cell release was accompanied by increased gelatinase/collagenase release from the biofilm following treatment with iron. Therefore, the results from this study suggest that elevated iron or norepinephrine in the presence of transferrin may result in enhanced degradation of host tissues in patients with advanced atherosclerosis. Current belief suggests that the action of MMPs produced by macrophages/foam cells, neutrophils, and smooth muscle cells causes the deterioration of the fibrous cap (49, 50); however, the results of this study indicate that infecting bacteria may also play a role.

**FIG 5:**
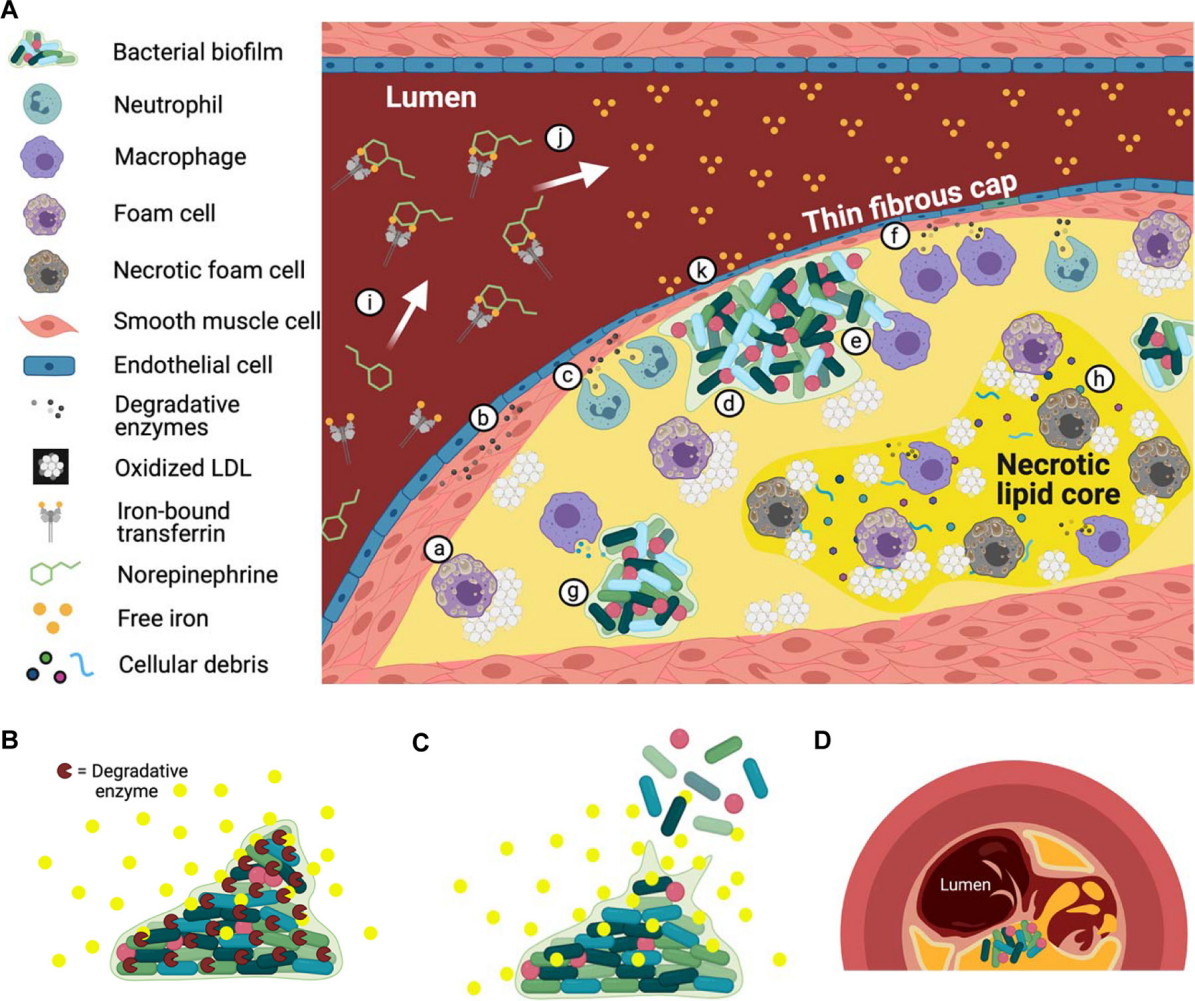
Proposed model of atherosclerosis and bacterial-driven plaque rupture during physiologic stress. Atherosclerotic lesions contain many different cell types and molecules that interact in various ways (A). Interactions include: (small letter a) transformation of macrophages into foam cells by engulfment of oxidized low-density lipoprotein (oxLDL); (letter b) reactive oxygen species (ROS) and degradative enzymes near the fibrous cap, released by smooth muscle cells; (letter c) neutrophils; and (letter f) macrophages. Interactions between bacterial biofilm cells and human cells in the plaque deposit (letter d) may attract phagocytosing macrophages (letter e), as well as ROS and degradative enzyme-releasing macrophages (letter g). The necrotic lipid core (letter h) which houses foam cells, enzymatically active macrophages, necrotic foam cells, oxLDL, and cellular debris. The model of plaque rupture proposed in this study depends on the physiologic stress response that results in the release of norepinephrine into the bloodstream, where it is met by transferrin (letter i). The interaction between NE and Tf causes the release of free iron into the blood (letter j). This increase in free iron can then be sensed by biofilm bacteria present near or along the fibrous cap (letter k). Biofilm bacteria may disperse and secrete degradative enzymes (B) to break through the matrix and allow cells to escape (C). These enzymes may cause collateral damage to the fibrous cap and result in plaque rupture (D). Figure was created with BioRender.com.

Previous work in our lab has demonstrated the presence of multispecies bacterial biofilm deposits near the fibrous cap (13). If bacteria at this location are dispersing in response to elevated levels of free iron in the blood as a result of the physiologic stress response, they could be releasing increased amounts of collagenases and gelatinases which ultimately degrade the fibrous cap and potentially lead to plaque rupture. We have proposed a model of atherosclerosis that includes the action of bacterial enzymes and their ability to influence the stability of arterial plaque deposits (Fig. 5). We recognize that the release of collagen-degrading enzymes may be specific to the artificial environment of our *in vitro* tests, and only indicates the potential for collagen degradation in patients with atherosclerosis. Further research is necessary to determine whether plaque-associated biofilm bacteria undergo dispersion *in vivo* and release enzymes which degrade the fibrous cap. The implication that bacteria may contribute to heart attack or stroke could result in drastic changes to diagnostic and treatment strategies and has the potential to improve cardiovascular outcomes associated with atherosclerosis.

## MATERIALS AND METHODS

### Bacterial strains and culture media.

Cutibacterium acnes VP1 (ATCC 6919) overnights were cultured from frozen stocks into 20 mL full strength RCM (BD, Franklin Lakes, NJ) supplemented with 0.1% sodium thioglycolate (Spectrum Chemical, New Brunswick, NJ) for 48 h. *C. acnes* cultures were used as inocula for biofilm culturing in 1/5 RCM with 0.1% sodium thioglycolate. *C. acnes* was isolated from *in vitro* three-species biofilms using RCM agar (Merck, Billerica, MA) supplemented with 30 μg/mL polymyxin B (TCI Chemicals, Portland, OR) and 15 μg/mL furazolidone (TCI Chemicals, Portland, OR).

Staphylococcus epidermidis ECNU-He1 was cultured from frozen stocks into 5 mL full strength RCM with 0.1% sodium thioglycolate for 24 h at 37°C with 5% CO_2_, then subcultured (using a 1% inoculum) into fresh 5 mL RCM with 0.1% sodium thioglycolate and grown for 16 to 18 h under the same conditions. Secondary S. epidermidis cultures were inoculated into 1/5 RCM with 0.1% sodium thioglycolate for biofilm culturing. S. epidermidis was isolated from *in vitro* three-species biofilms using Mannitol salts agar (BD, Franklin Lakes, NJ).

Pseudomonas aeruginosa PA14 overnights were cultured from frozen stocks into 5 mL full strength RCM for 24 h at 37°C with shaking at 220 rpm, then subcultured (using a 1% inoculum) into fresh 5 mL RCM and grown for 16 to 18 h under the same conditions. Secondary P. aeruginosa overnights were inoculated into 1/5 RCM for biofilm culturing. P. aeruginosa was isolated from *in vitro* three-species biofilms using Pseudomonas isolation agar (BD, Franklin Lakes, NJ).

Biofilms were grown in 2.75 mL 1/5 RCM with or without 0.1% sodium thioglycolate at 37°C with 5% CO_2_. Sodium thioglycolate was used to establish *C. acnes* and S. epidermidis, but upon the addition of P. aeruginosa, sodium thioglycolate was no longer used as a supplement in the medium.

### Three-species biofilm culturing.

A mixed consortium of *C. acnes*, S. epidermidis, and P. aeruginosa was grown as a biofilm (referred to as three-species biofilm) in semi-batch culture using tissue culture-treated 24-well plates (Corning Inc., Corning, NY). Approximately 7 × 10^3^ *C. acnes* VP1 cells per well were inoculated, and plates were incubated for 24 h. Medium from the wells was removed, leaving only *C. acnes* cells which had attached to the wells. Next, approximately 2 × 10^3^ S. epidermidis ECNU-He1 cells per well were inoculated and the plates were incubated for 6 h. Medium was then removed, allowing only attached *C. acnes* and S. epidermidis cells to remain. Approximately 5 × 10^5^ P. aeruginosa PA14 cells per well were inoculated and the plates were incubated for 6 h. At 36 h, the medium was removed, leaving only the cells that had adhered. Plates were incubated for a total of 5 days, and spent medium was exchanged and replenished with fresh medium at 48, 60, 72, 84, 96, 108, and 120 h.

### Treatment with FeSO_4_.

At 123 h postinoculation, wells were washed with phosphate buffer (1 mM KH_2_PO_4_ and 3.5 mM K_2_HPO_4_ [pH 7]), then the biofilms present in half of the wells were treated with 1/5 RCM as a control, and the other half were treated with 1/5 RCM supplemented with 0.1 mM FeSO_4_. Plates were incubated for 45 min at 37°C with 5% CO_2_. Following incubation, supernatants were extracted and cells from biofilms were collected separately by scraping and collection of the buffer, followed by a rinse step to collect any cells which remained attached to the well following scraping. Cells present in both the supernatant liquids and the collected biofilms were plated on selective agar to determine the CFU of cells released and cells that remained in the biofilm for each of the three species. Cell release was determined by calculating the percentage of cells present in the supernatant liquids compared to the total number of cells for each treatment. This calculation was performed on both a species level, to determine whether each individual organism underwent a dispersion response, and in total, combining counts from all three organisms to assess overall dispersion efficacy. This assay was performed in biological triplicate, and statistical analysis was done using a two-tailed *t* test.

### Treatment with norepinephrine and transferrin.

At 123 h postinoculation, biofilms were treated with 1/5 RCM supplemented with 0.4 mM NE (EMD Millipore, Burlington, MA) and 0.5 g/L Tf (Alfa Aesar, Ward Hill, MA) and the following controls: 1/5 RCM, 1/5 RCM supplemented with 0.4 mM NE, and 1/5 RCM with 0.5 g/L Tf. Released and biofilm cell collection, and calculations of cells released, were performed as described above. This experiment was run on biological triplicates and statistical analysis was performed by analysis of variance.

### Bulk liquid processing for enzyme assays.

Following plating, the supernatant liquids overlying biofilms treated with 1/5 RCM from 24 wells were collected and pooled. The bulk liquids from biofilms treated with 1/5 RCM with 0.1 mM FeSO_4_ were also combined. Bulk liquids were lyophilized according to the methods of Lanter and Davies (13). Briefly, cell-free spent medium was prepared by centrifugation of the bulk medium containing released cells at 12,000 × *g* for 5 min at 4°C. Then the overlying liquid fractions were syringe-filtered using 0.2-μm filters (Pall, Cortland, NY) to remove any remaining cellular debris. Samples were frozen at −20°C until further use. Frozen samples were lyophilized at −86°C and 0.320 mBar for 48 h (Labconco FreeZone Legacy 2.5 L, Labconco, Kansas City, MO). Lyophilized samples were stored at −20°C until they were used for enzyme assays. This was done to enrich the concentration of enzymes released by bacteria and improve detection of enzymatic activity.

### Lipase and protease activity assay.

This assay was performed as previously described by Lanter and Davies (13), with some modifications. Lyophilized cell-free spent-medium samples from 24 wells per treatment were resuspended in 500 μL 1× TE buffer. The samples treated with 0.1 mM FeSO_4_ were normalized based on the number of cells released in the 0 mM FeSO_4_ treatment. A 100-μL volume of each sample was loaded into wells measuring 6 mm in diameter which were punched into agar plates containing 25 mL skim milk agar (Becton, Dickinson and Co., Franklin Lakes, NJ) or tributyrin agar (HiMedia, Mumbai, India) substrates. Plates were parafilmed and incubated at 37°C for 24 h, at which point the diameter of the clearance zone was measured. Lyophilized sterile medium was used as a control to ensure that the degradation of the substrates observed was only due to the activity of extracellular enzymes released by the bacteria. This assay was performed in five biological replicates and statistical analysis was run using a two-tailed *t* test.

### Gelatinase and collagenase assay.

Lyophilized cell-free spent-medium samples were prepared as described above, with one modification: samples were resuspended in 500 μL Gelatinase buffer (0.15 M NaCl, 5 mM CaCl_2_, 0.2 mM NaN_3_, 50 mM Tris-HCl [pH 7.4]) and run using DQ Gelatin (Invitrogen, Carlsbad, CA), a fluorescently tagged gelatin substrate, according to the manufacturer’s recommendations. Concentrated samples were normalized to the number of cells released, as described previously, then diluted 10-fold to diminish the background fluorescence of the concentrated medium. A 100-μL volume of each sample was combined with an equal volume containing 100 μg/μL DQ Gelatin in clear-bottomed 96-well plates. Concentrated sterile media with and without iron were used as fluorescence controls to account for fluorescent properties of components of the medium. Plates were incubated at 37°C for 30 min with readings taken every 5 min using a fluorescence plate reader (SpectraMax i3x, Molecular Devices, San Jose, CA) with 495 nm laser excitation and 520 nm emission.

Relative fluorescence readings at each time point were used to generate enzyme activity curves. Linear slopes were calculated between 10 and 30 min for each trial, since the fluorescent readings demonstrated variability, diminishing the coefficient of determination (R^2^) within the first 10 min of readings. The slopes, or rates of gelatin hydrolysis, were compared against a standard curve to determine the total amount of collagenase present in each sample. The total enzyme concentrations for each replicate were then compared against the number of cells released in the respective trials to determine the amount of enzyme released per cell during dispersion versus that of the control biofilms.
