# Supplementary material for: Laboratory Grown Biofilms of Bacteria Associated with Human Atherosclerotic Carotid Arteries Release Collagenases and Gelatinases during Iron-Induced Dispersion
Source: Microbiol Spectr. 2022 May 11;10(3):e01001-21. doi: 10.1128/spectrum.01001-21 (PMC9241811; doi:10.1128/spectrum.01001-21)
Supplement: SUPPLEMENTAL FILE 1 — Supplemental material. Download spectrum.01001-21-s001.pdf, PDF file, 0.2 MB [file spectrum.01001-21-s001.pdf]

SUPPLEMENT

SUPPLEMENTAL MATERIALS AND METHODS

Multi-species biofilm development

Biofilm development was studied to ensure biofilms reached a stable consortium of all three microorganisms. Three-species biofilm development was determined by viable plate counts using selective growth media as listed above. At 24, 30, 36, 48, 72, 96 and 120 hr, two wells were sampled to determine colony forming units (CFU) of *C. acnes, S. epidermidis* and *P. aeruginosa*. Medium was removed, biofilms were washed with a phosphate buffered solution (1 mM KH_2PO_4 and 3.5 mM K_2HPO_4, pH 7) and 1 mL phosphate buffer was added to the wells for resuspension of biofilm cells. Cells were removed by scraping and collection of the buffer, followed by a rinse step to collect any cells that remained attached to the well following scraping. Samples were then diluted and plated on selective agar to determine the number of cells present in the biofilm at each time point (Supplemental Figure 1).

Growth curve with $FeSO_4$, norepinephrine and transferrin

Overnight cultures of *S. epidermidis* and *P. aeruginosa* were pre-equilibrated in 1/5 RCM for 3 hours to replicate the medium change prior to treatment of biofilms with $FeSO_4$ or norepinephrine and transferrin. Due to the slower growth rate of *C. acnes*, overnight cultures of *C. acnes* were pre-equilibrated in 1/5 RCM for 6 hours at 37°C with 5% CO_2 prior to taking OD_{600} readings. After pre-equilibration, cultures were subsectioned and supplemented with one of the following components: 0.1 mM $FeSO_4$, 0.4 mM norepinephrine, 0.5 g/L transferrin, 0.5 g/L transferrin with 0.4 mM norepinephrine, or an equal volume 1/5 RCM as a control. The treatment time used for multi-species biofilms was 45 min, so to ensure the increase in supernatant cell numbers was not due to increased growth rate due to the addition of iron, treatments were applied for 3 hours, four times longer than the treatment time. OD_{600} readings were acquired for all cultures at 0, 30, 60, 90, 120, 150 and 180 min, then converted to cell

number, and plotted against time. The growth rates were calculated and statistically compared. Experiments were performed in biological triplicate, and a representative trial was selected for each of the organisms (Supplemental figure 2).

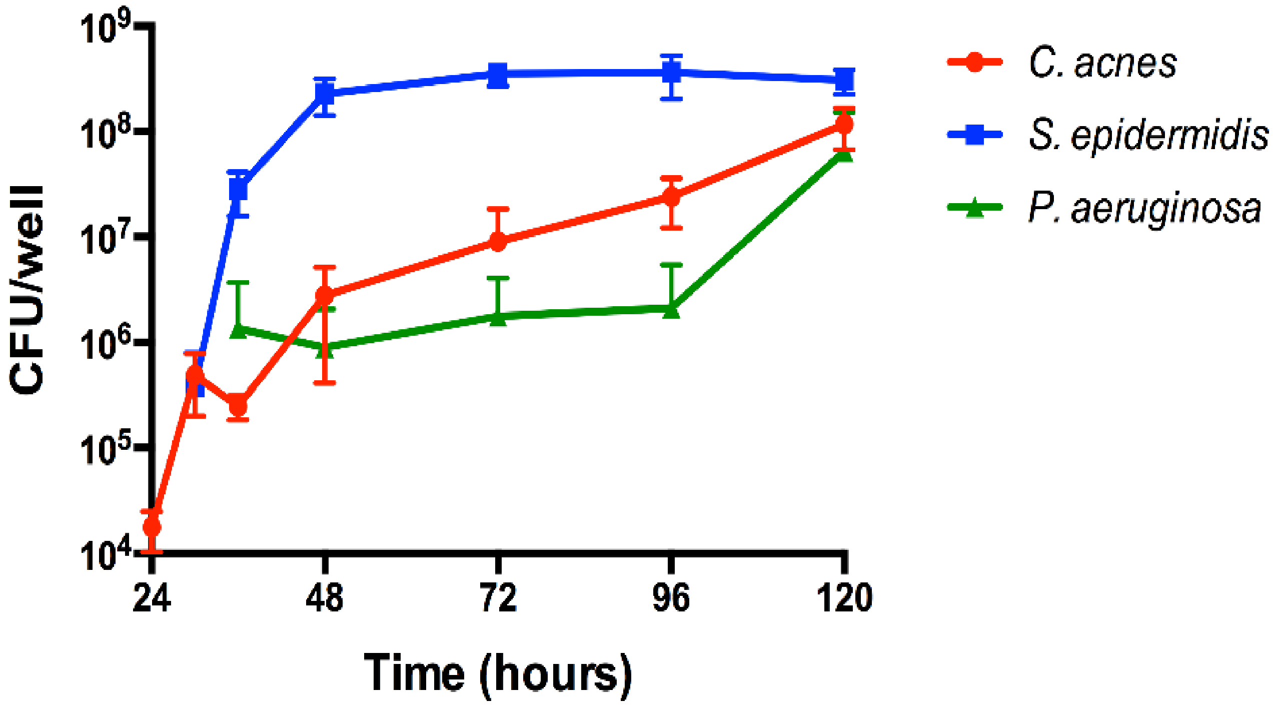

Supplemental Figure 1. Multi-species Biofilm Growth and Development. Time course representing cell number for respective species grown in tri-species biofilms. Biofilms were grown in 2.75 mL 1/5 RCM for 5 days in 24 well plates at 37°C with 5% CO_2 with medium changes every 12 hours. Approximately 6.65 x 10^3 *C. acnes* VP1 cells were inoculated at time zero and allowed to attach for 24 hours, at which time 1.75 x 10^3 *S. epidermidis* ECNU-He1 cells were inoculated and allowed to attach for 6 hours. At 30 hours, 4.65 x 10^5 *P. aeruginosa* PA14 cells were inoculated and allowed to attach for 6 hours. At 24, 36, 48, 72, 96 and 120 hours 2 wells were scraped and plated to determine colony forming unit (CFU) of the respective species at each timepoint (n=3). Error bars represent the standard deviation of cell counts performed at each time point.

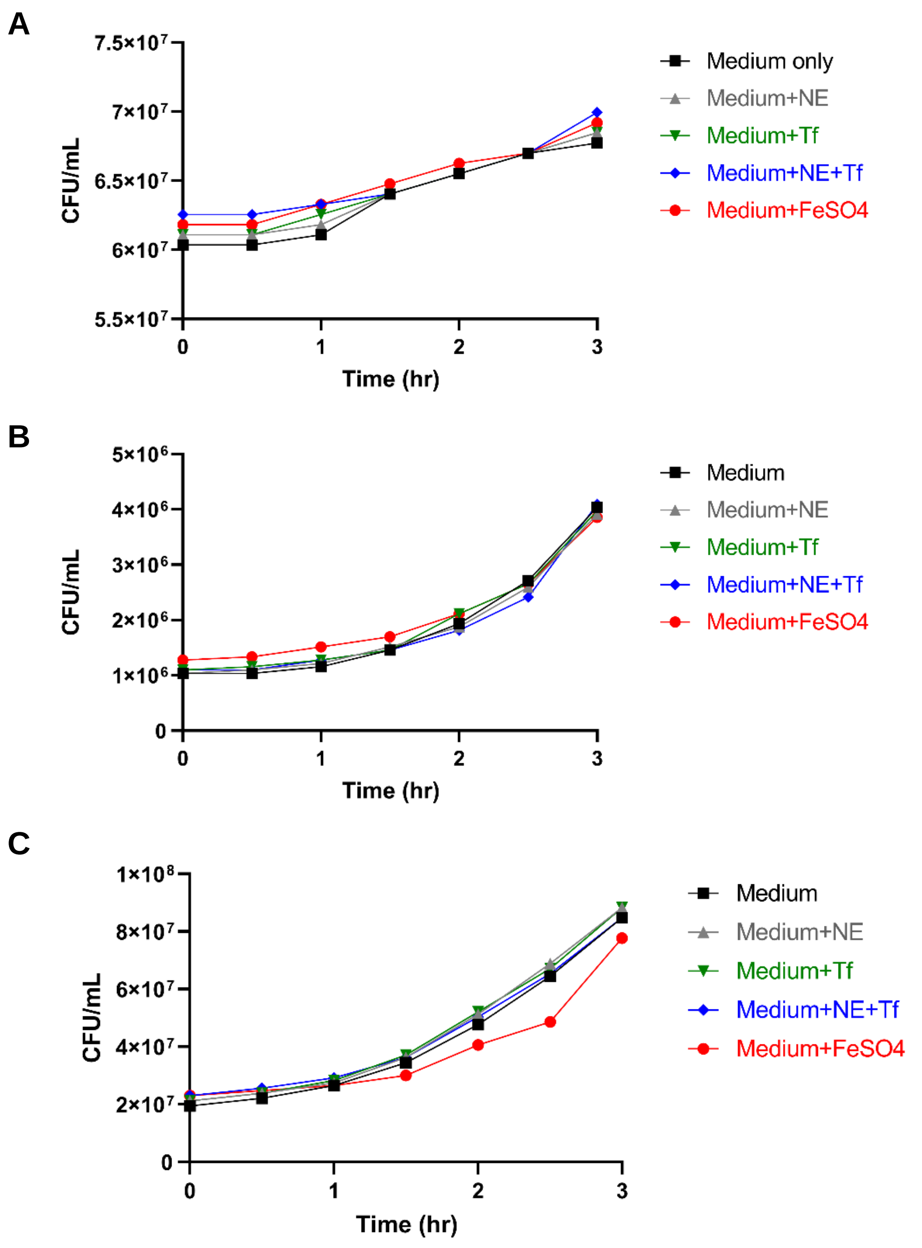

Supplemental Figure 2. Growth curves with medium supplements. Time course representing cell number for respective species grown in bulk media. Cultures were supplemented with 0.4 mM Norepinephrine (NE), 0.5 g/L Transferrin (Tf), 0.4 mM NE + 0.5 g/L Tf, 0.1 mM $FeSO_4$, or medium only as a control. The total volume for each culture was 25 mL, and they were grown for 3 hours at 37°C with 5% CO_2 with OD_{600} readings every 30 min. The OD_{600} readings were converted to cell number using a calibration curve relating OD_{600} to CFU for each of the three species. **(A)** *C. acnes* VP1. **(B)** *S. epidermidis* ECNU-He1. **(C)** *P. aeruginosa* PA14. Growth curves were performed on biological triplicates to calculate generation time and ensure the treatments did not increase the growth rate of each species. Growth rates were compared by ANOVA, and demonstrated no statistical significance. One representative dataset was graphed for each of the three organisms.
